# Efficacy and safety of tirofiban bridge as an alternative to suspension of dual antiplatelet therapy in patients undergoing surgery: a systematic review

**DOI:** 10.1590/1677-5449.210113

**Published:** 2021-12-01

**Authors:** Lorrane Vieira Siqueira Riscado, João Henrique Sendrete de Pinho, Armando de Carvalho Lobato

**Affiliations:** 1 Universidade Federal de Juiz de Fora – UFJF, Faculdade de Medicina – FAMED, Juiz de Fora, MG, Brasil.; 2 Instituto de Cirurgia Vascular e Endovascular de São Paulo – ICVE-SP, São Paulo, SP, Brasil.

**Keywords:** bridge, tirofiban, surgery

## Abstract

Use of a tirofiban bridge is an alternative to simply withdrawing dual antiplatelet therapy prior to operating on patients at high risk of stent thrombosis and bleeding. We aimed to evaluate the efficacy and safety of this protocol in patients undergoing surgery within 12 months of a percutaneous coronary intervention involving stenting. We performed a systematic review based on searches of the PubMed, Web of Science, Cochrane, Embase, Lilacs, and Scielo databases and of the references of relevant articles on the topic. Five of the 107 studies identified were included after application of eligibility criteria and analysis of methodological quality, totaling 422 patients, 227 in control groups. Notwithstanding the limitations reported, four of the five studies included indicate that the tirofiban bridge technique is effective for reducing adverse cardiac events and is safe in terms of not interfering with the risk of hemorrhagic events or bleeding. However, randomized clinical trials are needed to provide robust evidence.

## INTRODUCTION

Many patients are put on dual antiplatelet therapy (DAPT) with aspirin and P2Y12 receptor inhibitors with the objective of preventing stent thrombosis (ST) and adverse cardiovascular and cerebrovascular events such as death, myocardial infarction (MI), and stroke.^1^^-^ 3 This therapy is employed because the risk of perioperative ST is higher during the first 4 to 6 weeks after placement of both conventional and drug-eluting stents, so guidelines recommend use of DAPT for a minimum of 12 months after cardiac revascularization.^4^ Withdrawal of DAPT is a major risk factor for ST, particularly during this period.^5^^,^ 6

It is also reported that approximately 7% of the 3 million people who undergo stenting annually will need non-cardiac surgical interventions during the first year after percutaneous coronary intervention (PCI), which is the period during which it is necessary to keep them on DAPT.^7^ As a consequence, the increased risk of bleeding because of the antiplatelet drugs combined with the need for surgical procedures constitute a challenge for the cardiologists, anesthesiologists, and surgeons responsible for perioperative management of these patients.^8^

The strategy habitually employed was to suspend these drugs prior to surgery and reintroduce them after it had been confirmed that the patient was free from bleeding. However, recent evidence demonstrated that suspension is related to higher rates of ischemic cardiac events^9^^-^ 12 and death^13^ and is thus unacceptable, indicating a need to review this approach. It is also known that patients undergoing surgery are in a prothrombotic, inflammatory, and hypoxic state, especially those undergoing orthopedic, cardiac, and oncological surgery.^14^^,^ 15 Moreover, coronary stents constitute thrombogenic materials in a medium that facilitates hypercoagulation and the process of cracking the atheromatous plaque during angioplasty exposes pro-thrombotic substances, provoking platelet activation and adhesion.^16^^,^ 17 These are possible reasons for the high rates of morbidity and mortality among patients who undergo surgery after stenting.

However, data on management of these patients are varied. This is because there are no large-scale randomized studies and both guidelines and expert opinion recommend that cases should be analyzed individually to determine risk of ischemic cardiac events and of significant bleeding and also the potential consequences of delaying surgery (for example, progression of cancer).^18^ In this scenario, bridging with intravenous antiplatelet drugs has been proposed as a possible treatment option for patients at high risk of thrombosis and high risk of bleeding in the perioperative period.^18^^-^ 21

The objective of this article is to assess, by means of a systematic review of relevant publications available in the literature, the efficacy and safety of tirofiban bridge in patients with at risk of ST and of bleeding who need to suspend DAPT during the perioperative period.

## METHODOLOGY

This study is a systematic literature review conducted according to the Preferred Reporting Items for Systematic Reviews and Meta-Analyses (PRISMA) methodology.^22^ Since it employs secondary data, there was no need for submission to or approval by a Research Ethics Committee.

### Eligibility criteria

The PICO strategy was used to develop a research question to identify the best evidence and review the existing literature,^23^ as shown in Table 1. Our inclusion criteria were studies that assessed the efficacy of tirofiban bridge in patients who underwent surgery within 12 months of stent placement and were on DAPT but had to suspend it because of a high risk of bleeding. We excluded review articles, articles expressing expert opinion, duplicate articles, and articles unavailable for download.

**Table 1:** Components of the PICO23 strategy used to construct research questions and search for evidence.

Population	Patients who had undergone percutaneous coronary intervention (PCI) with stenting and were scheduled for surgery within 1 year of PCI while on dual antiplatelet therapy (DAPT)
Intervention	Tirofiban bridge with suspension of DAPT
Control	Patients who had DAPT suspended but were not put on a tirofiban bridge protocol
Outcome	Primary outcome: occurrence of adverse ischemic events Secondary outcome: occurrence of hemorrhagic events

### Search strategy

Studies were identified by searching electronic databases and analyzing the references of relevant articles. The databases used were PubMed, Cochrane, Literatura Latino-Americana e do Caribe em Ciências da Saúde (LILACS), Web of Science, EMBASE, and Scientific Electronic Library Online (SciELO). Searches were run for studies published up to May 2021 using the keywords ((bridge) AND (tirofiban) AND (surgery)), selected from the Descritores em Ciências da Saúde (DeCS) platform. No language restrictions were set.

### Methodological quality

The methodological quality of articles was analyzed using an 18-criteria checklist from the modified Delphi technique.^24^ Articles were reviewed by two authors individually. The maximum score for each study was 18 points, since each criterion is weighted equally. Studies were considered of acceptable quality if they achieved 9 or more positive answers (≥ 50%).

### Data extraction

Studies rated as of acceptable quality were compiled in a table for organization and extraction of data according to the following criteria: name of author, enrollment period, study design, total number of patients and number of patients who underwent tirofiban bridge, time elapsed between PCI and surgery, type of stent, type of surgery, and outcomes. The characteristics of the patients in each study were organized in a table containing number of patients, age, sex, smoking, systemic diseases, such as diabetes and arterial hypertension, use of beta blockers and/or statins, presence of peripheral vascular disease, and ejection fraction.

The specifications of the tirofiban bridge protocol used in each study were compiled in a table covering suspension of the P2Y12 inhibitor and/or aspirin, start of tirofiban infusion, quantity infused, withdrawal of tirofiban, and reintroduction of the P2Y12 inhibitor and/or aspirin.

### Analysis of the data

With the objective of evaluating the efficacy of tirofiban bridging, the primary outcome analyzed was occurrence of adverse ischemic events, such as ST, MI, and death. To evaluate the safety of tirofiban bridging, the secondary outcome analyzed was occurrence of hemorrhagic events.

## RESULTS

### Study selection

**Figure 1:**
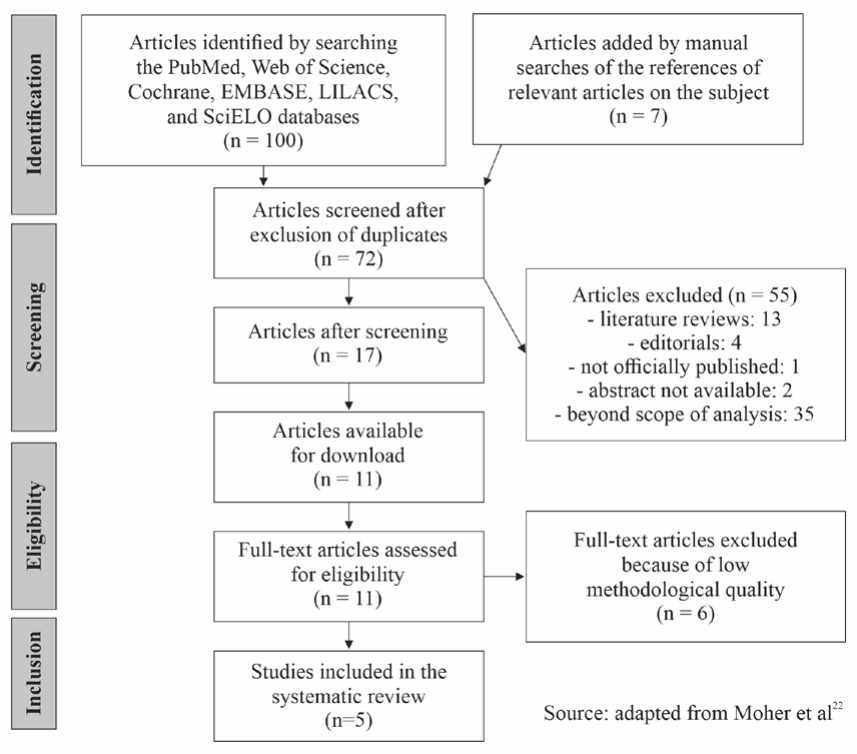
Flow diagram illustrating identification, screening, eligibility, and inclusion of studies in the systematic review. n = number of articles.

A total of 107 articles were identified, 72 of which remained after duplicates were excluded. After screening the articles by applying the inclusion and exclusion criteria, 11 articles remained for assessment of methodological quality.^25^^-^ 35 After analysis of methodological quality, five articles were included in the review.^26^^,^^28^^,^^31^^,^^34^ The search process and results are illustrated in Figure 1.

### Characteristics of the studies

Three of the five studies analyzed were conducted in Italy,^26^^,^^30^^,^^31^ one took place in Holland,^28^ and one was carried out in China.^34^ As can be observed in Table 2, only the article with the largest number of patients had a control group.^26^ In that study, 314 patients were enrolled, 87 of whom were put on the tirofiban bridge protocol, while the remaining 227, in whom DAPT was suspended without bridging, formed the control group.^26^ Overall, non-cardiac surgery predominated, both among patients who underwent bridging and among those in the control groups. There were 422 patients in the studies reviewed, 67.78% of whom underwent non-cardiac surgery. Death and MI were included in the primary outcome definition in all of the studies; ST was only listed as an outcome in two studies,^26^^,^^28^ but was reported in the results in the other three studies;^30^^,^^31^^,^^34^ revascularization of the target lesion or target-vessel was reported in two;^31^^,^^34^ and stroke was only reported in one study.^26^ Hemorrhagic events and bleeding were included in the secondary outcomes of four studies,^26^^,^^28^^,^^31^^,^^34^ regardless of the specific definition of each of them; two of the four studies^26^^,^^31^ used criteria from the Thrombolysis in Myocardial Infarction (TIMI)^36^ study to classify them.

**Table 2:** Data collected from studies selected for the systematic review.

**Reference**	**Enrollment period**	**Study design**	**Number of patients**	**Patients on tirofiban bridge protocol**	**Interval from PCI to surgery**	**Type of stent**	**Type of surgery**	**Outcomes**
De Servi et al.^26^	2007-2013	Observational, retrospective	314	87	Median of 104 days with interquartile range (IQR) of 5-365 days for patients on the tirofiban bridge protocol and 105 (IQR: 0–360) for the control group	Patients on the tirofiban bridge protocol: drug-eluting only (85%); metal only (9.2%); drug-eluting and metal (5.8%) of. In the control group: drug-eluting only (41.4%); metal only (54.2%); drug-eluting and metal (4.4%).p	Patients on the tirofiban bridge protocol: cardiac (32.2%) and non-cardiac (67.8%). In the control group patients: cardiac (27.8%) and non-cardiac (62.2%).	NACE^a^ at 30 days
Marcos et al.^28^	2006-2010	Observational, retrospective	36	36	Mean of 80 days with standard deviation of 66 days	Drug-eluting	Cardiac (42%); non-cardiac (58%)	MACE^b^ at 30 days; hemorrhagic events^c^ within 30 days
Polito et al.^30^	2012-2017	Observational, retrospective	21	21	Mean of 7.2 days with standard deviation of 3.2 days	Drug-eluting	Cardiac	Complications after surgery, clinical and echocardiographic status, death, reinfarction and/or cardiovascular and non-cardiovascular events over 21.6±15.6 months of follow-up
Savonitto et al.^31^	-	Observational, prospective	30	30	Median of 4 months with range of 1-12 months	Drug-eluting	Cardiac (30%); non-cardiac (70%)	Cardiovascular death, MI^d^, acute occlusion of the target-lesion demonstrated angiographically while in hospital and need for surgical re-exploration because of bleeding; safety of the treatment^e^
Xia et al.^34^	2011	Observational, prospective	21	21	Median of 6 months and range of 3-8 months	Drug-eluting	Non-cardiac	cardiovascular Death, MI and target-lesion revascularization from first to third month after hospital discharge; acute left ventricular failure, unstable angina pectoris, adverse hemorrhagic events and severe bleeding^f^ from first to third month after hospital discharge

MI = myocardial infarction; PCI = intervention coronary percutaneous.

a Net Adverse Clinical Events, defined as a combination of major adverse cardiac and cerebrovascular events (MACCE = mortality from all causes, myocardial infarction, definitive stent thrombosis, stroke) and significant bleeding, classified according to the criteria from the Thrombolysis in Myocardial Infarction (TIMI) study;^36^

b Major adverse cardiac events, defined as any death, repeat myocardial infarction, target-vessel revascularization, target-lesion revascularization, or stent thrombosis;

c Defined as hematuria, gastrointestinal bleeding, blood transfusion without bleeding, fall in hemoglobin concentration, or postoperative bleeding needing reintervention;

d Defined as clinical signs and symptoms of myocardial ischemia combined with elevation of creatine phosphokinase-MB (CK-MB) levels exceeding 3 times the normal limit;

e Assessed in terms of number of units of blood products transfused and non-operative bleeding, defined according to the criteria from the Thrombolysis in Myocardial Infarction (TIMI) study;^36^

f Intracranial bleeding manifesting with fall in Hb of ≥5 g/dL or fall in hematocrit of ≥15%.

Overall, among the 195 patients who were put on a tirofiban bridge protocol, there were two MI, one ST, 13 bleeding events, and no deaths. In the only controlled study,^26^ there were 12 MI, three ST, 36 bleeding events, and six deaths in the control group.

As shown in Table 3, age, sex and preexisting diseases were reported in all of the studies selected,^26^^,^^28^^,^^30^^,^^31^^,^^34^ Of the total of 422 patients, 325 (77%) were men and 97 (23%) were women. Comorbidities reported included 99 (23.46%) out of 422 patients with diabetes and 270 (63.98%) patients with systemic arterial hypertension (SAH).

**Table 3:** Characteristics of the patients in each of the studies selected.

**Characteristics**	**De Servi et al.** 26		**Marcos et al.** 28	**Polito et al.** 30	**Savonitto et al.** 31	**Xia et al.** 34
**Tirofiban bridge**	**Control**
Number of patients	87	227	36	21	30	21
Age	Median of 67.4 years and interquartile range of 25-83 years	Median of 69.2 years and interquartile range of 41-90 years	66±11 years (mean ± standard deviation)	62±9 years (mean ± standard deviation)	65 (25-80) years (mean ± standard deviation)	Mean of 63 years range of 45-74 years
Male	64 (73.6%)	180 (79.3%)	25 (69.4%)	20 (95.3%)	23 (76.7%)	13 (61.9%)
Female	23 (26.4%)	47 (20.7%)	11 (30.6%)	1 (0.7%)	7 (23.3%)	8 (38.1%)
Smoking	-	-	2 (5.6%)	12 (57.1%)	-	-
Diabetes	21 (24.1%)	55 (24.2%)	4 (11.1%)	6 (28.6%)	5 (16.7%)	8 (38.1%)
Systemic arterial hypertension	53 (60.9%)	168 (74.0%)	8 (22.2%)	13 (61.9%)	14 (46.7%)	14 (66.7%)
Taking beta blockers	67 (77.0%)	155 (68.3%)	-	-	21 (70%)	15 (71.4%)
Taking statins	71 (81.6%)	168 (74.0%)	-	-	23 (76.7%)	20 (95.3%)
Peripheral vascular disease	-	-	-	-	4 (13.3%)	5 (23.8%)
Ejection fraction	51.2±9.1% (mean ± standard deviation)	49.9±10.0% (mean ± standard deviation)	-	9 (42.9%): >55% 7 (33.3%): 45-55% 4 (19.0%): 35-45% 1 (4.8%): <35%	Median of 55% with range of 35-68%	-

Table 4 lists the specifications of the tirofiban bridge protocols used in each study. In all studies, the P2Y12 inhibitor was withdrawn 5 days before the surgical procedure. However, the studies differed in terms of suspension of aspirin. In two studies,^26^^,^^31^ aspirin was only withdrawn in cases involving laparotomy. In the study by Polito et al.,^30^ none of the patients had aspirin suspended, whereas in Xia et al.,^34^ aspirin was withdrawn 5 days before surgery.

**Table 4:** Characteristics of the tirofiban bridge protocol in each study.

**Characteristics**	**De Servi et al.** 26	**Marcos et al.** 28	**Polito et al.** 30	**Savonitto et al.** 31	**Xia et al.** 34
Withdrawal of the P2Y_12_ inhibitor	5 days before surgery	5 days before surgery	5 days before surgery	5 days before surgery	5 days before surgery
Withdrawal of aspirin	Not withdrawn except in laparotomy cases	Withdrawn in 19.4% of the patients, 5 days before surgery	Not withdrawn	Not withdrawn except in laparotomy cases	5 days before surgery
Start of tirofiban infusion	3 days before surgery	4±1.5 days before surgery (mean ± standard deviation)	3 days before surgery	4 days before surgery	4 days before surgery
Quantity infused	0.4 mg/kg/min for 30 minutes, then 0.1 mg/kg/min (the dose was reduced to 50% if creatinine clearance <30 mL/min)	-	0.1 µg/kg/min (the dose was reduced to 50% if creatinine clearance <30 mL/min)	0.4 mg/kg/min for 30 minutes, then 0.1 mg/kg/min (the dose was reduced to 50% if creatinine clearance <30 mL/min)	0.1 mg/kg/min
Withdrawal of tirofiban	4 hours before surgery (8 hours if creatinine clearance <30 mL/min)	4 hours before surgery	4 hours before surgery (8 hours if glomerular filtration rate ≤30 mL/min)	4 hours before surgery (8 hours if creatinine clearance <30 mL/min)	4 hours before surgery
Reintroduction of the P2Y_12_ inhibitor	As soon as patient able to accept oral administration again, with an attack dose of 300 mg and then 75 mg administered daily	12 to 24 hours after the intervention	Reintroduced as soon as possible	As soon as patient able to accept oral administration again, with an attack dose of 300 mg and then 75 mg administered daily	After surgery, with an attack dose of 300 mg and then 75 mg once a day
Reintroduction of aspirin	12 hours after the intervention with an IV injection of 250 mg of lysine acetylsalicylate once o day until the patient is able to resume oral administration, then 75 to 100 mg once a day orally	1.6±1.2 days after the intervention (mean ± standard deviation)	As soon as possible	12 hours after the intervention with an IV injection of 250 mg of lysine acetylsalicylate once o day until the patient is able to resume oral administration, then 75 to 100 mg once a day orally	After surgery with an initial dose of 300 mg, then 100 mg once a day

IV = intravenous.

The start of tirofiban infusion and the quantity infused varied according to each study protocol. However, in all of the studies analyzed, infusion was suspended 4 hours before surgery. Reintroduction of the P2Y12 inhibitor and aspirin also varied across the different studies.

### Methodological quality

All five of the articles included in the systematic review scored 50% or more for the 18 criteria from the modified Delphi technique^24^ for analysis of the internal quality of observational studies. The studies scored, in ascending order, 11, 13, 14, 14, and 17 points, with a mean methodological quality score of 13.8 points (76.7%). The quality criteria and the detailed scores for each of the 11 full text articles assessed for eligibility are shown in Table 5.

**Table 5:** Analysis of methodological quality using the modified Delphi technique.

**Criteria**	**Bona et al.** 25	**De Servi et al.** 26	**D’Urbano et al.** 27	**Marcos et al.** 28	**Park et al.** 29	**Polito et al.** 30	**Savonitto et al.** 31	**Vlachou et al.** 32	**Walker et al.** 33	**Xia et al.** 34	**Zhou et al.** 35
**Study objective**											
1. Is the hypothesis/aim/objective of the study clearly stated in the abstract, introduction or methods section?	YES	YES	YES	YES	YES	NO	YES	YES	YES	YES	YES
**Study population**											
2. Are the characteristics of the participants included in the study described?	YES	YES	YES	YES	YES	YES	YES	YES	YES	YES	YES
3. Were the cases collected in more than one centre?	NO	YES	NO	NO	NO	YES	YES	NO	NO	NO	NO
4. Are the eligibility criteria (inclusion and exclusion criteria) for entry to the study explicit and appropriate?	NO	YES	NO	YES	NO	YES	YES	NO	NO	YES	NO
5. Were participants recruited consecutively?	NO	YES	NO	YES	NO	NO	YES	NO	YES	YES	NO
6. Did participants enter the study at a similar point in the disease?	NO	NO	NO	NO	NO	NO	NO	YES	NO	NO	NO
**Intervention and co-intervention**											
7. Was the intervention clearly described in the study?	YES	YES	YES	YES	YES	YES	YES	YES	YES	YES	YES
8. Were additional interventions (co-interventions) clearly reported in the study?	YES	YES	NO	YES	NO	NO	YES	NO	NO	YES	YES
**Outcome measures**											
9. Are the outcome measures clearly defined in the introduction or methods section?	NO	YES	NO	YES	NO	YES	YES	NO	YES	YES	NO
10. Were relevant outcomes appropriately measured with objective and/or subjective methods?	NO	YES	NO	YES	NO	YES	YES	NO	YES	YES	NO
11. Were outcomes measured before and after intervention?	NO	YES	NO	YES	NO	YES	YES	NO	NO	YES	NO
**Statistical analysis**											
12. Were the statistical tests used to assess the relevant outcomes appropriate?	NO	YES	NO	NO	NO	NO	YES	NO	NO	YES	NO
**Results and conclusions**											
13. Was the length of follow-up reported?	YES	YES	YES	YES	YES	YES	NO	YES	NO	YES	NO
14. Was the loss to follow-up reported?	NO	YES	NO	NO	NO	NO	NO	NO	NO	NO	NO
15. Does the study provide estimates of the random variability in the data analysis of relevant outcomes?	NO	YES	NO	YES	NO	YES	YES	NO	NO	NO	NO
16. Are adverse events reported?	YES	YES	YES	YES	NO	YES	YES	YES	YES	YES	YES
17. Are the conclusions of the study supported by results?	YES	YES	NO	YES	NO	YES	YES	YES	YES	YES	NO
**Conflicts of interest and financing**										
18. Are both competing interest and source of support for the study reported?	YES	YES	NO	NO	NO	NO	NO	YES	NO	YES	NO
**Total**	8	17	5	13	4	11	14	8	8	14	5
											

### Primary and secondary outcomes

The results of the studies included are listed in Table 6. Four of the five studies demonstrated that tirofiban bridge has efficacy for reducing adverse ischemic events, to the extent that there were no ST in three studies.^28^^,^^31^^,^^34^ In the fourth,^30^ just one of 21 patients had an ST. None of these four studies reported deaths, MI, re-infarction, or revascularization of the target lesion.

**Table 6:** Results of the studies selected for the systematic review.

**Results**	**De Servi et al.** 26		**Marcos et al.** 28	**Polito et al.** 30	**Savonitto et al.** 31	**Xia et al.** 34
**Tirofiban bridge**	**Control**
Number of patients	87	227	36	21	30	21
Stent thrombosis	0	3	0	1	0	0
Myocardial infarction	2	12	0	0	0	0
Death	0	6	0	0	0	0
Bleeding	5	36	6	0	2	0

The only controlled study^26^ did not show a statistically significant effect of tirofiban bridge for reduction of major adverse cardiac events, with two patients presenting MI in the group that underwent tirofiban bridge. There were 12 MI, three ST, and six deaths in the control group in this study. No deaths or ST occurred in the group of patients on the tirofiban bridge protocol.

Four^26^^,^^30^^,^^31^^,^^34^ of the five studies assessed demonstrated the safety of tirofiban bridge, which did not impact on the occurrence of bleeding or hemorrhagic events. In two of these studies,^30^^,^^34^ there were no hemorrhagic or bleeding events. In the study with 30 patients,^31^ two patients exhibited bleeding during the postoperative period.

In the controlled study,^26^ tirofiban bridge was associated with a statistically significant reduction in the occurrence of intrahospital bleeding, reporting five major bleeding events in the group subjected to tirofiban bridge and 36 major bleeding events in the control group. In one of the studies assessed, six bleeding events occurring within 30 days of the surgical intervention were reported.^28^

## DISCUSSION

The data obtained in this systematic review of the literature suggest that the tirofiban bridge technique is feasible in patients with high risk of ST and of bleeding subjected to heart surgery or non-cardiac surgery and enables surgical procedures to be performed with lower rates of adverse ischemic events, without significantly affecting hemorrhagic events. Our results are compatible with a large systematic review of the literature published in 2014 in the journal Internal and Emergency Medicine, which assessed 420 patients who were put on a bridge protocol with glycoprotein IIb/IIIa inhibitors (eptifibatide or tirofiban) or with cangrelor. In the 2014 review, the bridge technique was effective 96.2% of the time (95% confidence interval 94.4 - 98.0%), with a 100% success rate for tirofiban and with 81.0% of 121 patients treated with tirofiban bridge therapy free from bleeding/transfusion events.^37^

A prospective observational cohort study published in 2012, enrolling 6,816 consecutive patients, demonstrated that withdrawal of clopidogrel during the first 6 months after placement of a drug-eluting stent was significantly related to ST during that period.^38^ In this context, intravenous administration of tirofiban can be proposed as an option for patients at high risk of ST, who would have DAPT withdrawn due to high risk of bleeding during the surgical procedure (for example, certain ophthalmologic surgeries, surgery of the middle ear, or neurosurgeries).^19^

In the present review, the majority of studies demonstrated clinical benefits of tirofiban bridging. In the controlled study, no deaths were reported among the 195 patients who underwent bridge therapy, whereas six deaths were reported in the group that only suspended DAPT. In all five studies, with a total of 195 patients treated with bridge therapy, only one case of ST and two MI cases were reported.

The reasoning underlying perioperative suspension of DAPT considers the increased likelihood of bleeding while in surgery and during the postoperative period if these medications are maintained. A meta-analysis of 41 studies with a total of 49,590 patients assessed the risk of surgical bleeding in patients on low-dose aspirin, demonstrating that aspirin multiplied the baseline bleeding rate of surgical procedures by a factor of 1.5 (1.0-2.5) without increase in surgical mortality or morbidity.^39^ In some of the studies analyzed, it was shown that maintenance of aspirin over the perioperative period in combination with the tirofiban bridge did not influence bleeding rates and in the study that reported six bleeding events,^28^ only one of the patients affected was taking aspirin. In the study that reported two bleeding events,^31^ only one patient was taking aspirin and suffered a bleeding event classified as minor by the TIMI study criteria,^36^ requiring transfusion due to preexisting anemia. The study by De Servi et al.^26^ did not mention whether patients who had bleeding were on aspirin or not and the decision on whether to discontinue it was made by the surgeon.

With regard to adverse cardiac events, there was only one case of ST in the 195 patients treated with tirofiban bridge and no deaths. Although De Servi et al.^26^ did not demonstrate a statistically significant effect for reduction of major adverse cardiac events, the patients in that study who underwent tirofiban bridge exhibited two MI compared to 12 MI in the control group and there were zero ST in bridge patients compared to three ST in the control group. There were also six deaths in the control group, whereas there were no deaths in the bridge group. Some limitations may have affected the results of this article, considering that there was no randomization of patients and that treatment depended on decisions made by the surgeons at the hospitals at which the patients were treated on whether to adopt treatment with a tirofiban bridge and whether to withdraw clopidogrel only or to withdraw both clopidogrel and aspirin.

Even though the time elapsed between stenting and the subsequent surgical procedure was relatively short, at 2 to 6 months, no major adverse cardiac events were reported in the study by Marcos et al.^28^ In turn, Savonitto et al.^31^ demonstrated the efficacy of tirofiban bridge therapy, since none of their patients exhibited an ischemic cardiac event during the perioperative period. Xia et al.^34^ also positively demonstrated the efficacy of tirofiban bridge therapy, since there were no ischemic cardiac events. Although Polito et al.^30^ described one case of ST, they emphasized that the tirofiban bridge prevented a fatal thrombotic event, since angioplasty was performed and there were no deaths, re-infarctions, or any other cardiovascular events during the 21.6±15.6 months of follow-up.

With regard to hemorrhagic or bleeding events, Marcos et al.^28^ reported six hemorrhagic events, two of which occurred after reintroduction of clopidogrel and aspirin, and the severity of bleeding was not considered high in the majority of patients. De De Servi et al.^26^ reported that the tirofiban bridge protocol reduced intrahospital bleeding to a statistically significant extent, considering that the patients were subjected to surgery involving a high risk of bleeding. Indeed, the low rates of death, MI, ST, and stroke were achieved without increasing the risk of bleeding among these patients.

Although Savonitto et al.^31^ reported two bleeding events, they did not exceed what is commonly accepted for the types of surgery to which the patients were subjected. The patient who had an event classified as minor bleeding according to the TIMI criteria^36^ had undergone endoscopic bladder surgery and had preexisting anemia; while the patient who had an event classified as major bleeding had undergone a hemicolotomy and suffered proctorrhagia on the seventh postoperative day, after reintroduction of clopidogrel.

Xia et al.^34^ demonstrated the safety of tirofiban bridge therapy during the perioperative period and over a 3-month follow-up, during which no hemorrhagic or bleeding events were reported. Polito et al.^30^ also demonstrated the safety of the technique, since they had no hemorrhagic or bleeding events and just three cases of uncomplicated anemia.

It is worth pointing out that each study had a different follow-up period after surgery and tirofiban infusion. While some studies followed patients for 1 month, others did so for 3 to 21 months or only conducted intrahospital monitoring until patient discharge. As such, the outcome periods assessed were reasonably different between the studies, which could have affected the results.

Furthermore, even though the secondary outcome was positive in the majority of the studies and all the patients included had been classified as at high risk of bleeding, calculating the bleeding risk index is complex and must account not only for the risk intrinsic to the surgery, but also for the patient’s individual risk, which was not clearly explained in detail in the studies. Therefore, if the risk was not calculated appropriately, taking a multidisciplinary approach, the non-interference of tirofiban bridging in the risk of hemorrhagic and bleeding events may have been overestimated.

Moreover, it is important to consider these results with caution, since favorable results could have occurred because of other factors, such as use of beta blockers and statins during the perioperative period, and also because the number of patients treated is small. Another possible factor involved in the favorable outcomes is that the tirofiban bridge protocol involves more detailed monitoring of the patient during the perioperative period, since infusion is started 3 to 4 days before surgery and is only discontinued 4 hours before the procedure. Therefore, the longer hospital stay and monitoring may be related to lower rates of bleeding, although the published data suggest that tirofiban infusion really does not interfere in the risk of bleeding during surgery.^40^

It should be pointed out that this review is subject to limitations. One of these is that the majority of data were extracted from retrospective studies. Another limitation is the different definitions of ischemic and hemorrhagic events adopted by the studies. The results could also have been affected by the heterogeneous nature of populations, the different protocols used, and the study designs, since many characteristics related to the patients were not reported in all of the articles analyzed, limiting our analysis.

Moreover, in the controlled study,^26^ there were more patients with SAH in the control group, which could have contributed to the higher number of adverse events in that group compared to the group of patients who underwent the tirofiban bridge protocol, considering that this comorbidity is one of the principal risk factors for cardiovascular events. Longitudinal data obtained in the Framingham Heart Study indicated that people with SAH have twice the relative risk of cardiovascular disease compared to normotensive people.^41^

There was also variability in the different periods of time elapsed between PCI and surgery, which is an important predictor of ischemic cardiac events.^38^ Other differences observed were the time at which tirofiban infusion was initiated, the quantity infused, and the time at which the P2Y12 inhibitor and aspirin were reintroduced, which may also be related to occurrence or not of hemorrhagic and ischemic cardiac events.

While there are limitations related to the lack of randomized clinical trials, use of glycoprotein IIb/IIIa inhibitors during the perioperative period in patients at high risk of ST and high risk of bleeding is recommended by guidelines and by experts.^18^^-^ 20 ^,^ 42 ^,^ 43 We therefore believe that this study contributes additional useful information to the current literature. It should be also be pointed out that it is very unlikely that controlled and randomized studies will be conducted testing the safety and efficacy of tirofiban bridge therapy in comparison to discontinuation of the thienopyridine derivative with or without discontinuing aspirin, with no other strategies combined with suspension of treatment, considering the elevated number of adverse events and deaths in the control group in the study analyzed in this review.

## CONCLUSIONS

Patients who undergo surgery during the first 12 months after stenting are at increased risk of thrombotic events, indicating a need to reassess the practice of perioperative suspension of antiplatelet drugs. In cases in which patients are at high risk of ST and of hemorrhagic events, a tirofiban bridge appears to be a safe and effective strategy for management of perioperative medication, taking into account the reduction in the likelihood of adverse ischemic without increasing the likelihood of hemorrhagic events. However, more robust evidence is needed. Regardless, it is always recommended that these patients receive multidisciplinary management.
