# Supplementary material for: Efficacy and safety of tirofiban bridge as an alternative to suspension of dual antiplatelet therapy in patients undergoing surgery: a systematic review
Source: J Vasc Bras. 2021 Dec 1;20:e20210113. doi: 10.1590/1677-5449.210113 (PMC8668084; doi:10.1590/1677-5449.210113)
Supplement: Translation

# Translation: Eficácia e segurança da ponte de tirofiban como alternativa à suspensão da terapia antiplaquetária dupla em pacientes submetidos à cirurgia: uma revisão sistemática

**Authors:** Lorrane Vieira Siqueira Riscado, João Henrique Sendrete de Pinho, Armando de Carvalho Lobato

**Affiliations:** 1 Universidade Federal de Juiz de Fora – UFJF, Faculdade de Medicina – FAMED, Juiz de Fora, MG, Brasil.; 2 Instituto de Cirurgia Vascular e Endovascular de São Paulo – ICVE-SP, São Paulo, SP, Brasil.

**Keywords:** ponte, tirofiban, cirurgia

## Abstract

A ponte de tirofiban é uma alternativa à suspensão da terapia antiplaquetária dupla no perioperatório de pacientes com alto risco de trombose de stent e de sangramento. Objetivamos avaliar a eficácia e a segurança deste protocolo em pacientes submetidos à cirurgia em até 12 meses após intervenção coronária percutânea com stent. Realizamos uma revisão sistemática por meio de pesquisa nas bases PubMed, Web of Science, Cochrane, EMBASE, LILACS e SciELO e nas referências de artigos relevantes ao tema. Dos 107 trabalhos encontrados, cinco foram incluídos após análise dos critérios de elegibilidade e da qualidade metodológica, totalizando 422 pacientes, sendo 227 do grupo controle. Apesar das limitações reportadas, quatro dos cinco estudos incluídos indicam que a ponte de tirofiban é eficaz em reduzir eventos cardíacos adversos e segura ao não interferir no risco de eventos hemorrágicos ou sangramentos. Todavia, são necessários ensaios clínicos randomizados para evidências robustas.

## INTRODUÇÃO

Um grande número de pacientes utiliza a terapia antiplaquetária dupla (TAPD) com ácido acetilsalicílico (AAS) e inibidores do receptor P2Y12 com o intuito de prevenir trombose de stents (TS) e eventos adversos cardiovasculares e cerebrovasculares, como morte, infarto do miocárdio (IM) e acidente vascular cerebral (AVC)^1^^-^ 3. Isso ocorre porque o risco de TS no período perioperatório, tanto para stents convencionais quanto para farmacológicos, é maior nas primeiras 4 a 6 semanas após o implante, sendo recomendado por diretrizes a utilização da TAPD por, no mínimo, 12 meses após revascularização cardíaca^4^. Descontinuar a TAPD, principalmente nesse período, é um grande fator de risco para TS^5^^,^ 6.

Relata-se, ainda, que aproximadamente 7% dos 3 milhões de pacientes submetidos anualmente a implante de stent requerem intervenção cirúrgica não cardíaca dentro do primeiro ano de realização da intervenção coronária percutânea (ICP), período em que ainda há a necessidade de manutenção da TAPD^7^. Dessa forma, devido ao uso dos antiplaquetários, o aumento do risco de sangramento nos procedimentos cirúrgicos representa um desafio para cardiologistas, anestesiologistas e cirurgiões no manejo perioperatório desses pacientes^8^.

A suspensão desses fármacos previamente à cirurgia e o seu reinício após a comprovação de ausência de sangramentos costumava ser a estratégia utilizada. Todavia, evidências recentes demonstram que a suspensão está relacionada com maiores taxas de eventos cardíacos isquêmicos^9^^-^ 12 e morte^13^, tornando-a, portanto, inaceitável e indicando a necessidade de revisão dessa conduta. Também é sabido que o paciente submetido à cirurgia está em estado pró-trombótico, inflamatório e de hipóxia, especialmente no que tange a cirurgias ortopédicas, cardíacas e oncológicas^14^^,^ 15. Ademais, stents coronários comportam materiais trombogênicos em um meio com facilidade de hipercoagulação, e o processo de rompimento da placa ateromatosa na angioplastia expõe substâncias pró-trombóticas, gerando ativação e adesão de plaquetas^16^^,^ 17. Essas podem ser as razões pelas quais há altas morbidade e mortalidade em pacientes submetidos à cirurgia após implante de stent.

Outrossim, os dados acerca do manejo desses pacientes têm se mostrado heterogêneos. Isso ocorre porque faltam grandes estudos randomizados, e as diretrizes e opiniões de especialistas recomendam que haja análise individual dos riscos de eventos cardíacos isquêmicos, de sangramentos significativos e de potenciais consequências do adiamento de cirurgias (por exemplo, progressão de câncer)^18^. Nesse cenário, a terapia de ponte com fármacos antiplaquetários intravenosos tem sido indicada como uma possível opção terapêutica para pacientes com alto risco trombótico e alto risco de sangramentos no período perioperatório^18^^-^ 21.

O objetivo deste artigo é avaliar, por meio de uma revisão sistemática de trabalhos relevantes disponíveis na literatura, a eficácia e a segurança da ponte de tirofiban em pacientes com alto risco de TS e de sangramento que necessitam suspender a TAPD no período perioperatório.

## METODOLOGIA

O estudo é uma revisão sistemática da literatura conduzida de acordo com a metodologia *Preferred Reporting Items for Systematic Reviews and Meta-Analyses* (PRISMA)^22^. Por utilizarmos dados secundários, não foi necessária submissão ou aprovação do Comitê de Ética em Pesquisa para o desenvolvimento do trabalho.

### Critérios de elegibilidade

Para identificação das melhores evidências e revisão da literatura existente, foi construída uma pergunta de pesquisa apropriada usando a estratégia PICO^23^, como demonstrado na Tabela 1. Nossos critérios de inclusão foram estudos que avaliaram a eficácia da ponte de tirofiban em pacientes submetidos à cirurgia em até 12 meses após o implante de stent em uso de TAPD que precisasse ser suspendida devido a alto risco de sangramento. Excluímos artigos de revisão, artigos de opinião de especialistas, artigos duplicados e artigos não disponíveis para *download*.

**Tabela 1:** Componentes da estratégia PICO23 para a construção de perguntas de pesquisa e busca de evidências.

População	Pacientes submetidos à intervenção coronária percutânea (ICP) com stent que se apresentam para cirurgia em uso de terapia antiplaquetária dupla (TAPD) em até 1 ano após a ICP
Intervenção	Ponte de tirofiban com suspensão da TAPD
Controle	Pacientes em que houve suspensão da TAPD sem aplicação do protocolo de ponte de tirofiban
Desfecho	Desfecho primário: ocorrência de evento isquêmico adverso Desfecho secundário: ocorrência de evento hemorrágico

### Estratégia de pesquisa

Os estudos foram identificados por meio de pesquisa eletrônica em bases de dados e análise das referências de artigos relevantes. As bases de dados utilizadas foram PubMed, Cochrane, Literatura Latino-Americana e do Caribe em Ciências da Saúde (LILACS), Web of Science, EMBASE e Scientific Electronic Library Online (SciELO). Foram pesquisados estudos publicados até maio de 2021 com os descritores ((bridge) AND (tirofiban) AND (surgery)) obtidos na plataforma Descritores em Ciências da Saúde (DeCS). Foram buscados artigos em todos os idiomas.

### Qualidade metodológica

Para analisar a qualidade metodológica dos artigos, utilizamos uma *checklist* de 18 critérios da técnica de Delphi modificada^24^. Os artigos foram revisados individualmente por dois autores. A pontuação máxima para cada estudo foi de 18 pontos, já que cada critério recebeu o mesmo peso. Os estudos considerados de qualidade aceitável foram aqueles que atingiram 9 ou mais respostas positivas (≥ 50%).

### Extração de dados

Os estudos considerados de qualidade aceitável foram agrupados em uma tabela para sistematização e extração dos dados, de acordo com os seguintes critérios: nome do autor, período de inclusão, desenho de estudo, número total de pacientes e número de pacientes submetidos a ponte de tirofiban, tempo entre ICP e cirurgia, tipo de stent, tipo de cirurgia e desfechos. As características dos pacientes de cada estudo foram organizadas em uma tabela contendo quantidade de pacientes, idade, gênero, tabagismo, doenças sistêmicas, como diabetes e hipertensão arterial, uso de betabloqueador e/ou estatinas, presença de doença vascular periférica e valor da fração da ejeção.

As especificações do protocolo ponte de tirofiban de cada estudo foram sistematizadas por meio de uma tabela abrangendo descontinuação do inibidor P2Y12 e/ou do AAS, início da infusão de tirofiban, quantidade infundida, descontinuação do tirofiban e retomada do inibidor P2Y12 e/ou do AAS.

### Análise dos dados

Com o objetivo de avaliar a eficácia da ponte de tirofiban, o desfecho primário analisado foi a ocorrência de eventos isquêmicos adversos, como TS, IM e morte. Para avaliar a segurança da ponte de tirofiban, o desfecho secundário analisado foi a ocorrência de eventos hemorrágicos.

## RESULTADOS

### Seleção dos estudos

Foram identificados 107 artigos, dos quais restaram 72 após a exclusão de duplicatas. Após a triagem com aplicação dos critérios de inclusão e exclusão, 11 artigos tiveram a qualidade metodológica avaliada^25^^-^ 35. Após a análise de qualidade metodológica, cinco artigos foram incluídos na revisão^26^^,^^28^^,^^31^^,^^34^. A representação esquemática do resultado da busca encontra-se na Figura 1.

**Figura 1:** Fluxograma de identificação, triagem, elegibilidade e inclusão de estudos na revisão sistemática. n = número de artigos.

### Características dos estudos

Dos cinco estudos analisados, três foram realizados na Itália^26^^,^^30^^,^^31^, um foi realizado na Holanda^28^ e um foi realizado na China^34^. Conforme a Tabela 2, apenas o artigo com maior quantidade de pacientes tinha grupo controle^26^. Nesse estudo, dos 314 pacientes, 87 foram submetidos ao protocolo de ponte de tirofiban, enquanto os 227 restantes, que apenas suspenderam a TAPD, corresponderam ao controle^26^. No total, houve uma predominância de cirurgia não cardíaca tanto no grupo submetido à ponte quanto no grupo controle, sendo que, dos 422 pacientes da revisão, 67,78% foram submetidos a cirurgia não cardíaca. Na definição do desfecho primário, morte e IM foram incluídos em todos os estudos; TS foi relatada como desfecho apenas em dois^26^^,^^28^, sendo reportada no resultado dos outros três estudos^30^^,^^31^^,^^34^; revascularização da lesão-alvo ou vaso-alvo foi reportada em dois^31^^,^^34^; e AVC foi relatado em apenas um estudo^26^. Entre os desfechos secundários, eventos hemorrágicos e sangramentos foram incluídos em quatro estudos^26^^,^^28^^,^^31^^,^^34^, a despeito da definição específica de cada um deles, sendo que dois dos quatro estudos^26^^,^^31^ utilizam critérios do ensaio *Thrombolysis in Myocardial Infarction* (TIMI)^36^ para classificação.

**Tabela 2:** Sistematização de dados dos estudos selecionados para revisão sistemática.

**Referência**	**Período de inclusão**	**Desenho do estudo**	**Número de pacientes**	**Pacientes submetidos à ponte de tirofiban**	**Tempo entre ICP e cirurgia**	**Tipo de stent**	**Tipo de cirurgia**	**Desfechos**
De Servi et al.^26^	2007-2013	Observacional, retrospectivo	314	87	Mediana de 104 dias e intervalo interquartil de (5-365) dias para os pacientes submetidos à ponte de tirofiban e 105 (0–360) para o grupo controle	Apenas farmacológico (85%); apenas metálico (9,2%); farmacológico e metálico (5,8%) dos pacientes submetidos à ponte de tirofiban. No grupo controle, apenas farmacológico (41,4%); apenas metálico (54,2%); farmacológico e metálico (4,4%) do grupo controle	Cardíaca (32,2%) e não cardíaca (67,8%) nos pacientes submetidos à ponte de tirofiban. Cardíaca (27,8%) e não cardíaca (62,2%) nos pacientes do grupo controle	MACE^a^ em 30 dias
Marcos et al.^28^	2006-2010	Observacional, retrospectivo	36	36	Média de 80 dias e desvio padrão de 66 dias	Farmacológico	Cardíaca (42%); não cardíaca (58%)	MACE^b^ em 30 dias; eventos hemorrágicos^c^ em 30 dias
Polito et al.^30^	2012-2017	Observacional, retrospectivo	21	21	Média de 7,2 dias e desvio padrão de 3,2 dias	Farmacológico	Cardíaca	Complicações após cirurgia, estado clínico e ecocardiográfico, morte, reinfarto e/ou eventos cardiovasculares e não cardiovasculares em 21,6±15,6 meses de acompanhamento
Savonitto et al.^31^	-	Observacional, prospectivo	30	30	Mediana de 4 meses e intervalo de (1-12) meses	Farmacológico	Cardíaca (30%); não cardíaca (70%)	Morte cardiovascular, IM^d^, demonstração angiográfica de oclusão aguda da lesão-alvo durante a hospitalização e necessidade de reexploração cirúrgica por causa de sangramento; segurança da terapia^e^
Xia et al.^34^	2011	Observacional, prospectivo	21	21	Mediana de 6 meses e intervalo de (3-8) meses	Farmacológico	Não cardíaca	Morte cardiovascular, IM e revascularização da lesão-alvo entre primeiro e terceiro mês de alta hospitalar; insuficiência ventricular esquerda aguda, angina pectoris instável, eventos adversos hemorrágicos e sangramentos graves^f^ entre o primeiro e terceiro mês de alta hospitalar

IM = infarto do miocárdio; ICP = intervenção coronária percutânea.

a Eventos clínicos adversos líquidos, definidos como a combinação de eventos cardiocerebrovasculares (MACE = morte por todas as causas, infarto do miocárdio, trombose de stent definitiva, derrame) e sangramentos significativos, classificados de acordo com os critérios do ensaio *Thrombolysis in Myocardial Infarction* (TIMI)^36^;

b Eventos cardíacos adversos maiores, definidos como qualquer morte, infarto miocárdico repetido, revascularização de vaso-alvo, revascularização de lesão-alvo ou trombose de stent;

c Definidos como hematúria, sangramentos gastrointestinais, transfusão de sangue sem sangramento, diminuição da concentração de hemoglobina ou sangramento pós-operatório que requeira nova intervenção;

d Definido como sinais clínicos e sintomas de isquemia miocárdica junto com aumento dos níveis de creatinoquinase-MB (CK-MB) maior que 3 vezes o limite normal;

e Avaliada em termos de número de unidades de constituintes sanguíneos transfundidos e sangramento não operatório, definido de acordo com os critérios do ensaio *Thrombolysis in Myocardial Infarction* (TIMI)^36^;

f Sangramento intracraniano manifesto com redução de Hb de ≥5 g/dL ou hematócrito de ≥15%.

No total, entre os 195 pacientes submetidos à ponte de tirofiban, foram relatados dois IM, um TS, 13 sangramentos e nenhuma morte. Já no único estudo controlado^26^, foram reportados 12 IM, três TS, 36 sangramentos e seis mortes no grupo controle.

De acordo com a Tabela 3, idade, sexo e doenças preexistentes foram características relatadas em todos os estudos selecionados^26^^,^^28^^,^^30^^,^^31^^,^^34^, sendo que, dos 422 pacientes, 325 (77%) eram homens e 97 (23%) eram mulheres. Em relação às comorbidades, 99 (23,46%) dos 422 pacientes tinham diabetes. Já quanto à hipertensão arterial sistêmica (HAS), 270 (63,98%) pacientes apresentavam a doença.

**Tabela 3:** Características dos pacientes de cada estudo selecionado.

**Características**	**De Servi et al.** 26		**Marcos et al.** 28	**Polito et al.** 30	**Savonitto et al.** 31	**Xia et al.** 34
**Ponte de tirofiban**	**Controle**
Número de pacientes	87	227	36	21	30	21
Idade	Mediana de 67,4 anos e intervalo interquartil de (25-83) anos	Mediana de 69,2 anos e intervalo interquartil de (41-90) anos	66±11 anos (média ± desvio padrão)	62±9 anos (média ± desvio padrão)	65 (25-80) anos (média ± desvio padrão)	Média de 63 anos e Intervalo de (45-74) anos
Masculino	64 (73,6%)	180 (79,3%)	25 (69,4%)	20 (95,3%)	23 (76,7%)	13 (61,9%)
Feminino	23 (26,4%)	47 (20,7%)	11 (30,6%)	1 (0,7%)	7 (23,3%)	8 (38,1%)
Tabagismo	-	-	2 (5,6%)	12 (57,1%)	-	-
Diabetes	21 (24,1%)	55 (24,2%)	4 (11,1%)	6 (28,6%)	5 (16,7%)	8 (38,1%)
Hipertensão arterial sistêmica	53 (60,9%)	168 (74,0%)	8 (22,2%)	13 (61,9%)	14 (46,7%)	14 (66,7%)
Uso de betabloqueador	67 (77,0%)	155 (68,3%)	-	-	21 (70%)	15 (71,4%)
Uso de estatinas	71 (81,6%)	168 (74,0%)	-	-	23 (76,7%)	20 (95,3%)
Doença vascular periférica	-	-	-	-	4 (13,3%)	5 (23,8%)
Fração de ejeção	51,2±9,1% (média ± desvio padrão)	49,9±10,0% (média ± desvio padrão)	-	9 (42,9%): >55% 7 (33,3%): 45-55% 4 (19,0%): 35-45% 1 (4,8%): <35%	Mediana de 55% com intervalo de (35-68%)	-

As especificações do protocolo de terapia ponte de tirofiban utilizadas em cada estudo estão demonstradas na Tabela 4. Em todos os estudos, a descontinuação do inibidor de P2Y12 ocorreu 5 dias antes do procedimento cirúrgico. Já quanto à descontinuação da aspirina, houve diferenças entre os estudos, sendo que em dois deles^26^^,^^31^ só foi realizada em caso de laparotomia. No estudo de Polito et al.^30^, nenhum paciente descontinuou a aspirina, enquanto, em Xia et al.^34^, a descontinuação de aspirina ocorreu 5 dias antes da cirurgia.

**Tabela 4:** Especificações do protocolo de terapia com ponte de tirofiban de cada estudo selecionado.

**Características**	**De Servi et al.** 26	**Marcos et al.** 28	**Polito et al.** 30	**Savonitto et al.** 31	**Xia et al.** 34
Descontinuação do inibidor P2Y_12_	5 dias antes da cirurgia	5 dias antes da cirurgia	5 dias antes da cirurgia	5 dias antes da cirurgia	5 dias antes da cirurgia
Descontinuação da AAS	Não ocorreu, exceto em caso de laparotomia	Ocorreu em 19,4% dos pacientes, sendo 5 dias antes da cirurgia	Não ocorreu	Não ocorreu, exceto em caso de laparotomia	5 dias antes da cirurgia
Início da infusão de tirofiban	3 dias antes da cirurgia	4±1,5 dias antes da cirurgia (média ± desvio padrão)	3 dias antes da cirurgia	4 dias antes da cirurgia	4 dias antes da cirurgia
Quantidade infundida	0,4 mg/kg/min durante 30 minutos, seguido por 0,1 mg/kg/min (a dose era reduzida em 50% se o *clearance* de creatinina fosse <30 mL/min)	-	0,1 µg/kg/min (a dose era reduzida em 50% se o *clearance* de creatinina fosse <30 mL/min)	0,4 µ/kg/min durante 30 minutos, seguido de 0,1 µg/kg/min (a dose era reduzida em 50% se o *clearance* de creatinina fosse <30 mL/min)	0,1 mg/kg/min
Descontinuação do tirofiban	4 horas antes da cirurgia (8 horas no caso de *clearance* de creatinina <30 mL/min)	4 horas antes da cirurgia	4 horas antes da cirurgia (8 horas no caso de taxa de filtração glomerular ≤30 mL/min)	4 horas antes da cirurgia (8 horas no caso de *clearance* de creatinina <30 mL/min)	4 horas antes da cirurgia
Retomada do inibidor P2Y_12_	Assim que o paciente pudesse retomar a administração oral, sendo uma dose de ataque de 300 mg e posteriormente 75 mg de administração diária	12 a 24 horas após a intervenção	Retomar tão cedo quanto possível	Assim que o paciente pudesse retomar a administração oral, sendo uma dose de ataque de 300 mg e posteriormente 75 mg de administração diária	Após a cirurgia, sendo uma dose de ataque de 300 mg, posteriormente 75 mg uma vez ao dia
Retomada da AAS	12 horas após a intervenção como uma injeção IV de 250 mg de salicilato de lisina acetil uma vez ao dia até que o paciente possa retomar a administração oral, seguindo com 75 a 100 mg uma vez ao dia por via oral	1,6±1,2 dias após a intervenção (média ± desvio padrão)	Retomar tão cedo quanto possível	12 horas após a intervenção como uma injeção IV de 250 mg de salicilato de lisina acetil uma vez ao dia até que o paciente possa retomar a administração oral, seguindo com 75 a 100 mg uma vez ao dia por via oral	Após a cirurgia, sendo uma dose inicial de 300 mg, posteriormente 100 mg uma vez ao dia

AAS = aspirina; IV = intravenosa.

O início da infusão de tirofiban e a quantidade infundida variaram de acordo com o protocolo de cada estudo. No entanto, em todos os estudos analisados, a infusão foi interrompida 4 horas antes da cirurgia. A retomada do inibidor de P2Y12 e da aspirina também apresentou variação entre os estudos.

### Qualidade metodológica

Os cinco artigos incluídos para revisão sistemática obtiveram 50% ou mais dos 18 critérios da técnica de Delphi modificada^24^ para análise de qualidade interna em estudos observacionais. As pontuações dos estudos, da menor para a maior, foram as seguintes: 11, 13, 14, 14 e 17, com média de 13,8 pontos (76,7%) em qualidade metodológica. Os critérios e a pontuação detalhada de cada artigo completo acessado para elegibilidade estão organizados na Tabela 5.

**Tabela 5:** Análise da qualidade metodológica pela técnica de Delphi modificada.

**Critérios**	**Bona et al.** 25	**De Servi et al.** 26	**D’Urbano et al.** 27	**Marcos et al.** 28	**Park et al.** 29	**Polito et al.** 30	**Savonitto et al.** 31	**Vlachou et al.** 32	**Walker et al.** 33	**Xia et al.** 34	**Zhou et al.** 35
**Objetivo do estudo**											
1. A hipótese/objetivo do estudo está claramente presente no resumo, na introdução ou na metodologia?	SIM	SIM	SIM	SIM	SIM	NÃO	SIM	SIM	SIM	SIM	SIM
**População do estudo**											
2. As características dos participantes incluídos são descritas no estudo?	SIM	SIM	SIM	SIM	SIM	SIM	SIM	SIM	SIM	SIM	SIM
3. Os casos foram coletados em mais de um centro?	NÃO	SIM	NÃO	NÃO	NÃO	SIM	SIM	NÃO	NÃO	NÃO	NÃO
4. Os critérios de elegibilidade (critérios de inclusão e exclusão) para entrada no estudo estão explícitos e apropriados?	NÃO	SIM	NÃO	SIM	NÃO	SIM	SIM	NÃO	NÃO	SIM	NÃO
5. Os participantes foram recrutados consecutivamente?	NÃO	SIM	NÃO	SIM	NÃO	NÃO	SIM	NÃO	SIM	SIM	NÃO
6. Os participantes entraram no estudo em um ponto similar da doença?	NÃO	NÃO	NÃO	NÃO	NÃO	NÃO	NÃO	SIM	NÃO	NÃO	NÃO
**Intervenção e co-intervenção**											
7. A intervenção foi claramente descrita no estudo?	SIM	SIM	SIM	SIM	SIM	SIM	SIM	SIM	SIM	SIM	SIM
8. Intervenções adicionais (co-intervenções) foram claramente reportadas no estudo?	SIM	SIM	NÃO	SIM	NÃO	NÃO	SIM	NÃO	NÃO	SIM	SIM
**Medida do desfecho**											
9. As formas de medir o desfecho estão claramente definidas na introdução ou na metodologia do estudo?	NÃO	SIM	NÃO	SIM	NÃO	SIM	SIM	NÃO	SIM	SIM	NÃO
10. Os desfechos relevantes foram medidos apropriadamente com métodos objetivos e/ou subjetivos?	NÃO	SIM	NÃO	SIM	NÃO	SIM	SIM	NÃO	SIM	SIM	NÃO
11. Os desfechos foram medidos antes e depois da intervenção?	NÃO	SIM	NÃO	SIM	NÃO	SIM	SIM	NÃO	NÃO	SIM	NÃO
**Análise estatística**											
12. A análise estatística utilizada para avaliar os desfechos relevantes foi apropriada?	NÃO	SIM	NÃO	NÃO	NÃO	NÃO	SIM	NÃO	NÃO	SIM	NÃO
**Resultados e conclusões**											
13. O período de acompanhamento foi reportado?	SIM	SIM	SIM	SIM	SIM	SIM	NÃO	SIM	NÃO	SIM	NÃO
14. A perda de acompanhamento foi reportada?	NÃO	SIM	NÃO	NÃO	NÃO	NÃO	NÃO	NÃO	NÃO	NÃO	NÃO
15. O estudo fornece estimativas da variabilidade aleatória na análise de dados de desfechos relevantes?	NÃO	SIM	NÃO	SIM	NÃO	SIM	SIM	NÃO	NÃO	NÃO	NÃO
16. Os eventos adversos são reportados?	SIM	SIM	SIM	SIM	NÃO	SIM	SIM	SIM	SIM	SIM	SIM
17. As conclusões do estudo são sustentadas pelos resultados?	SIM	SIM	NÃO	SIM	NÃO	SIM	SIM	SIM	SIM	SIM	NÃO
**Conflitos de interesse e financiamento**										
18. Os conflitos de interesse e as fontes de financiamento do estudo são reportados?	SIM	SIM	NÃO	NÃO	NÃO	NÃO	NÃO	SIM	NÃO	SIM	NÃO
**Total**	8	17	5	13	4	11	14	8	8	14	5

### Desfechos primário e secundário

Os resultados dos estudos avaliados estão representados na Tabela 6. Quatro dos cinco estudos avaliados demonstraram a eficácia da ponte de tirofiban na redução de eventos isquêmicos adversos, sendo que, em três estudos^28^^,^^31^^,^^34^, não houve TS. Em um deles^30^, apenas um dos 21 pacientes apresentou TS. Nesses quatro estudos, não foram reportados casos de morte, IM, reinfarto ou revascularização de lesão-alvo.

**Tabela 6:** Resultados dos estudos selecionados para revisão sistemática.

**Resultados**	**De Servi et al.** 26		**Marcos et al.** 28	**Polito et al.** 30	**Savonitto et al.** 31	**Xia et al.** 34
**Ponte de tirofiban**	**Controle**
Número de pacientes	87	227	36	21	30	21
Trombose de stent	0	3	0	1	0	0
Infarto do miocárdio	2	12	0	0	0	0
Morte	0	6	0	0	0	0
Sangramentos	5	36	6	0	2	0

No único estudo controlado^26^, a ponte de tirofiban não mostrou efeito estatisticamente significativo na redução de eventos cardíacos adversos maiores, com dois pacientes apresentando IM no grupo submetido à ponte de tirofiban. No grupo controle desse estudo, foram reportados 12 IM, três TS e seis mortes. Não foram relatados morte ou TS no grupo submetido à ponte de tirofiban.

Quatro^26^^,^^30^^,^^31^^,^^34^ dos cinco estudos avaliados demonstraram a segurança da ponte de tirofiban em não interferir na ocorrência de sangramentos ou eventos hemorrágicos. Em dois desses estudos^30^^,^^34^, não houve nenhum sangramento ou evento hemorrágico. Já no estudo com 30 pacientes^31^, dois pacientes apresentaram sangramento no pós-operatório.

No estudo controlado^26^, a ponte de tirofiban reduziu de forma estatisticamente significativa a ocorrência de sangramentos intra-hospitalares, sendo reportados cinco sangramentos maiores no grupo submetido à ponte de tirofiban e 36 sangramentos maiores no grupo controle. Foram reportados seis sangramentos em até 30 dias após a intervenção cirúrgica em um dos estudos avaliados^28^.

## DISCUSSÃO

Os dados obtidos nesta revisão sistemática da literatura sugerem que a ponte de tirofiban em pacientes com alto risco de TS e de sangramento submetidos a cirurgia cardíaca ou não cardíaca é factível e permite que os procedimentos cirúrgicos sejam realizados com menores taxas de eventos isquêmicos adversos, sem afetar significativamente os eventos hemorrágicos. Nossos resultados são condizentes com uma grande revisão sistemática da literatura publicada em 2014 na revista *Internal and Emergency Medicine*, que avaliou 420 pacientes submetidos a protocolo de ponte com inibidores da glicoproteína IIb/IIIa (eptifibatide ou tirofiban) ou com cangrelor. Na revisão de 2014, a técnica ponte foi eficaz 96,2% das vezes (intervalo de confiança de 95% 94,4 - 98,0%), sendo que a taxa de sucesso foi de 100% para o tirofiban, que também apresentou ausência de eventos de sangramento/transfusão em 81,0% dos 121 pacientes que foram submetidos à ponte com esse medicamento^37^.

Em um estudo prospectivo observacional de coorte publicado em 2012, que incluiu 6.816 pacientes consecutivos, foi demonstrado que a descontinuação de clopidogrel nos primeiros 6 meses após a implantação de stent farmacológico estava significativamente relacionada à TS nesse período^38^. Nesse contexto, a administração intravenosa de tirofiban pode ser proposta como alternativa para um paciente com alto risco de TS, que descontinuará a TAPD devido ao alto risco de sangramento no procedimento cirúrgico (por exemplo, determinadas cirurgias oftalmológicas, do ouvido médio ou neurocirurgias)^19^.

Na presente revisão, a maior parte dos estudos demonstrou benefícios clínicos da ponte de tirofiban. Não foi relatada morte nos 195 pacientes submetidos à ponte, enquanto seis mortes foram reportadas no grupo que apenas suspendeu a TAPD do estudo controlado. Em todos os cinco estudos, totalizando 195 pacientes submetidos à ponte, foram relatados apenas um caso de TS e dois de IM.

O raciocínio por trás da suspensão da TAPD no perioperatório leva em consideração a maior chance de sangramentos na cirurgia e no pós-operatório caso essas medicações sejam mantidas. Uma metanálise de 41 estudos, totalizando 49.590 pacientes, a qual avaliou o risco de sangramento cirúrgico em usuários de AAS em baixas doses, demonstrou que o AAS multiplicou a taxa de sangramento basal dos procedimentos cirúrgicos por um fator de 1,5 (1,0-2,5) sem que houvesse aumento de mortalidade ou morbidade cirúrgicas^39^. Em alguns dos estudos que analisamos, demonstrou-se que a continuidade da aspirina no período perioperatório juntamente com a realização da ponte de tirofiban não influenciou nas taxas de sangramentos, sendo que, no estudo que reportou seis sangramentos^28^, apenas um dos pacientes estava em uso de aspirina. No estudo que reportou dois sangramentos^31^, apenas um paciente estava em uso de aspirina e apresentou um sangramento classificado como menor pelo ensaio TIMI^36^ devido à anemia preexistente, requerendo transfusão. Já no estudo de De Servi et al.^26^, não foi mencionado se os pacientes que apresentaram sangramentos estavam ou não em uso de aspirina, sendo que a decisão de descontinuar o medicamento ficou à critério do cirurgião.

Quanto aos eventos cardíacos adversos, houve apenas um caso de TS nos 195 pacientes submetidos à ponte de tirofiban e nenhuma morte. Apesar de De Servi et al.^26^ não terem demonstrado efeito estatisticamente significativo na redução de eventos cardíacos adversos maiores, nesse artigo, os pacientes submetidos à ponte de tirofiban apresentaram dois IM contra 12 IM do grupo controle, bem como zero TS contra três TS do grupo controle. Também houve seis mortes no grupo controle do estudo, enquanto não houve nenhuma morte no grupo submetido à ponte. Algumas limitações podem ter afetado os resultados desse artigo, tendo em vista que não houve randomização dos pacientes e o tratamento dependeu da disposição dos cirurgiões e dos hospitais nos quais os pacientes foram tratados em aceitar ou não a terapia com ponte de tirofiban e suspender apenas o clopidogrel ou clopidogrel e aspirina.

No estudo de Marcos et al.^28^, apesar do tempo relativamente curto entre a colocação do stent e o procedimento cirúrgico, cerca de 2 a 6 meses, nenhum evento cardíaco adverso maior foi reportado. Já Savonitto et al.^31^ demonstraram a eficácia da ponte de tirofiban, pois nenhum dos pacientes apresentaram evento cardíaco isquêmico no período perioperatório. Xia et al.^34^ também demonstraram de forma positiva a eficácia da ponte de tirofiban, tendo em vista que não houve eventos cardíacos isquêmicos. Apesar de Polito et al.^30^ relatarem um caso de TS, destaca-se que a ponte de tirofiban preveniu um evento fatal de trombose, haja vista que foi realizada angioplastia e não houve morte, reinfarto ou quaisquer eventos cardiovasculares no seguimento de 21,6±15,6 meses.

Quanto à ocorrência de eventos hemorrágicos ou sangramentos, Marcos et al.^28^ relataram seis eventos hemorrágicos, sendo que dois ocorreram após o reinício do clopidogrel e da aspirina e a severidade do sangramento não foi considerada alta na maioria dos pacientes. Já De Servi et al.^26^ reportaram que a ponte de tirofiban reduziu de forma estatisticamente significativa os sangramentos intra-hospitalares, considerando que os pacientes foram submetidos a cirurgias de alto risco de sangramento. Ademais, as baixas taxas de morte, IM, TS e AVC foram atingidas sem aumento do risco de sangramento nesses pacientes.

Apesar de Savonitto et al.^31^ reportarem dois eventos de sangramento, estes não excederam o que é comumente aceito para os tipos de cirurgias às quais os pacientes foram submetidos: o paciente que apresentou um evento classificado como sangramento menor pelo ensaio TIMI^36^ havia sido submetido a uma cirurgia de bexiga endoscópica e possuía anemia preexistente; já o paciente que apresentou um evento classificado como sangramento maior havia sido submetido a uma hemicolectomia e apresentou proctorragia no sétimo dia do pós-operatório, após retomar o clopidogrel.

Xia et al.^34^ demonstrou a segurança da terapia com ponte de tirofiban no período perioperatório e no seguimento de 3 meses, nos quais não foi reportado nenhum evento hemorrágico ou sangramento. Em Polito et al.^30^, também foi demonstrada a segurança da terapia, tendo em vista que não houve nenhum evento hemorrágico ou sangramento, apenas três casos de anemia não complicada.

É válido ressaltar que cada estudo apresentou um tempo diferente de seguimento dos pacientes após a cirurgia e infusão de tirofiban. Enquanto alguns estudos fizeram o acompanhamento por 1 mês, outros fizeram por 3 até 21 meses ou apenas realizaram seguimento intra-hospitalar até a alta do paciente. Desse modo, os períodos dos desfechos avaliados foram razoavelmente diferentes entre os estudos, o que pode ter afetado os resultados.

Além disso, ainda que o desfecho secundário tenha sido positivo na maioria dos estudos e que todos os pacientes incluídos tenham sido classificados como de alto risco de sangramento, o cálculo desse índice é complexo e deve incluir não apenas o risco intrínseco da cirurgia, mas também o risco individual do paciente, o que não foi claramente detalhado nos estudos abordados. Desse modo, caso o cálculo do risco não tenha sido realizado de modo seguro e com abordagem multiprofissional, a não interferência da ponte de tirofiban no risco de sangramentos e eventos hemorrágicos pode ter sido superestimada.

Ademais, é importante considerar esses resultados de forma cautelosa, tendo em vista que os resultados favoráveis podem ter ocorrido por outros fatores, como uso de betabloqueadores e estatinas no perioperatório, além do pequeno número de pacientes tratados. Outro fator possível para os desfechos favoráveis é que o protocolo ponte de tirofiban está relacionado com um acompanhamento mais minucioso do paciente no perioperatório, uma vez que a infusão se inicia entre 3 a 4 dias antes da cirurgia, sendo descontinuada apenas 4 horas antes do procedimento. Desse modo, o maior tempo de hospitalização e monitoramento pode estar relacionado às menores taxas de ocorrência de sangramentos, apesar de dados da literatura sugerirem que a infusão de tirofiban realmente não interfere no risco de sangramento na cirurgia^40^.

Deve-se destacar que esta revisão apresenta limitações. Uma delas é o fato de que a maioria dos dados foram extraídos de estudos retrospectivos. Outra limitação é representada pelas diferentes definições de eventos isquêmicos e hemorrágicos adotadas por cada estudo. Os resultados também podem ser afetados devido à heterogeneidade de populações, dos diferentes protocolos utilizados e dos desenhos de estudo, sendo que, em relação aos pacientes, diversas características não foram relatadas em todos os artigos avaliados, limitando nossa análise.

Outrossim, no estudo controlado^26^ houve mais pacientes com HAS no grupo controle, o que pode ter contribuído para o maior número de eventos adversos nesse grupo em comparação aos pacientes submetidos à ponte de tirofiban, tendo em vista que essa comorbidade é um dos principais fatores de risco para eventos cardiovasculares. Dados longitudinais obtidos do *Framingham Heart Study* indicaram que portadores de HAS possuem um aumento maior que duas vezes no risco relativo de doença cardiovascular em comparação com normotensos^41^.

A variabilidade também advém dos diferentes intervalos de tempo entre a ICP e a cirurgia, que é um importante preditor de eventos cardíacos isquêmicos^38^. Outras diferenças observadas são o momento de início da infusão de tirofiban, a quantidade infundida e o momento de retomada do inibidor de P2Y12 e de AAS, que também podem estar relacionados à ocorrência ou não de eventos cardíacos isquêmicos e hemorrágicos.

Ainda que existam limitações relacionadas à falta de ensaios clínicos randomizados, o uso de inibidores de glicoproteína IIb/IIIa no perioperatório de pacientes com alto risco de TS e alto risco de sangramento é recomendado por diretrizes e por especialistas^18^^-^ 20 ^,^ 42 ^,^ 43. Portanto, acreditamos que este estudo acrescente informações úteis à literatura atual. Deve-se ressaltar ainda que estudos randomizados e controlados que testem a segurança e eficácia da ponte de tirofiban em oposição à descontinuação do tienopiridínico com ou sem descontinuação de AAS, sem outras estratégias associadas à interrupção da terapia, são pouco prováveis de serem executados, tendo em vista o elevado número de eventos adversos e mortes no grupo controle do estudo avaliado.

## CONCLUSÃO

Pacientes submetidos a cirurgia nos primeiros 12 meses após implante de stent possuem maior risco de eventos trombóticos, indicando a necessidade de rever a conduta perioperatória de suspensão de medicamentos antiplaquetários. Nos casos dos pacientes com alto risco de TS e de eventos hemorrágicos, a ponte de tirofiban parece ser uma estratégia eficaz e segura para o manejo da medicação no perioperatório, tendo em vista a redução na chance de ocorrência de eventos isquêmicos adversos sem aumento da chance de ocorrência de eventos hemorrágicos. No entanto, são necessárias evidências mais robustas. Ademais, a abordagem multiprofissional desses pacientes deve ser sempre preconizada.
